# Advantages of 3D printed patient-individual moulds in brachytherapy for facial skin cancer

**DOI:** 10.1007/s00066-025-02372-5

**Published:** 2025-02-26

**Authors:** Jörg Licher, Julia Achenbach, Janett Köhn, Markus Diefenhardt, Maximilian Fleischmann, Claus Rödel, Nikolaos Tselis, Ulla Ramm, Christian Scherf

**Affiliations:** 1 Goethe University Frankfurt, University Hospital, Department of Radiotherapy and Oncology, Theodor-Stern-Kai 7, 60590 Frankfurt am Main, Germany; 2 German Cancer Research Center (DKFZ), Heidelberg, Germany; 3 Partner Site Frankfurt am Main, German Cancer Consortium (DKTK), Frankfurt, Germany; 4 Frankfurt Cancer Institute, Frankfurt, Germany

**Keywords:** Skin cancer, Radiotherapy, Brachytherapy, Individual moulds, 3D printing

## Abstract

**Purpose:**

Facial skin cancer of 42 elderly frail patients was treated with individualised 3D-printed mould applicators for high-dose-rate (HDR) brachytherapy. The dosimetric outcome was compared to conventionally manufactured individual moulds used before.

**Methods:**

Tumour-adapted HDR brachytherapy source paths were pre-planned and dosimetrically optimised in the brachytherapy treatment planning system (TPS) using computed tomography (CT) data and considered in the design of the patient-individual moulds. Dosimetric outcome for the planning target volumes and organs at risk were statistically evaluated and compared for pre-planning, final clinical treatment planning with TG-43 formalism and retrospective tissue, material and CT density related TG-186 calculations.

**Results:**

Pre-planning allows reliable brachytherapy source paths design to achieve intended dosimetric clinical goals. The 3D-printed patient-specific moulds show a clear advantage in the dosimetric coverage of the target volume (improving D_90_ from 98.3% to 104.3%) and the protection of the relevant organs at risk (reduction up to 30% of maximum Dose). With the 3D-printed moulds only minor deviations were observed for TG-43 and TG-186 dose recalculations of the treated plans.

**Conclusion:**

Customised 3D printed moulds offer a safe and efficient technique to treat facial skin cancer in critical locations and complex clinical situations with HDR brachytherapy. The two-step planning process results in reliable PTV dose coverage and efficient sparing of eye lenses and eyeballs. Dosimetric outcome and interfractional position reproducibility with 3D printed moulds were superior to conventionally manufactured facial moulds with respect to the clinical goals.

## Introduction

High-dose-rate brachytherapy (HDR brachytherapy) has been a well-established method for treating skin tumours for many years. Techniques with different types of surface applicators are mentioned in several international guidelines [1–4]. They address various approaches, i.e. interstitial skin, electronic and HDR brachytherapy with commercial superficial applicators or flaps or with custom-made surface mould applicators. Regarding most facial skin cancer localisations, the planning target volumes (PTVs) are generally curved and difficult to cover, and relevant organs at risk (OARs) like eye lenses and the eyeballs are located close to the clinical PTV and must be spared. These preconditions can best be satisfied with custom-made mould-based brachytherapy applicators that are widely used for large patient collectives [5–7].

Our institution applies HDR brachytherapy with individually adapted surface moulds in the case of perinasal and periorbital tumour localisations for the large collective of frail elderly patients who are not eligible for primary surgery or definitive external-beam radiotherapy because of comorbidities or unfavourable tumour locations [8, 9]. The first clinical experiences were gained in 2019, with individual surface moulds made by a collaborating epithetician with glued-on plastic brachytherapy needles [8]. These are referred to below as conventionally manufactured moulds.

These experiences pointed out the limitations of this conventional technique and flowed into a project to establish 3D printing of patient-individual moulds to treat facial skin cancer of a wide range of different manifestations and localisations. Unlike for the conventional moulds externally manufactured by the epithetician, the standardised 3D manufacturing workflow offers PTV- and OAR-based catheter path optimisation (pre-planning) as an initial goal of mould design. This means that the source dwell positions are now adapted to the dosimetric plan requirements instead of adapting the isodoses of the treatment plans to the arbitrarily defined catheter paths of the moulds fabricated in advance. Clinical outcomes of the first patients were published recently and showed that 3D printed mould brachytherapy is a well-tolerated and safe treatment option [9].

In the meantime, the feasibility of 3D printing of individualised skin brachytherapy applicators has also become a focus of several other research groups. Currently, this method has passed through the evolution process from feasibility studies to routine clinical application [7, 9–12]. Workflows with systematic computed tomography (CT)-based mould design with dosimetric pre-planning prior to mould production have been developed. Advanced approaches and results of groups in different continents and countries are found in the very recent literature [9, 11–15]. It has been shown that treatment planning is reliable, and the correct and reproducible fit of the 3D printed moulds on the skin has been confirmed [11–15]. At the current stage of development, 3D printing has great potential as an alternative to conventional mould manufacturing techniques.

In the current report, we present the first systematic dosimetric assessment of the patient collective treated since 2021 with in-house-optimised and 3D printed moulds in direct comparison with the results obtained with conventionally manufactured moulds in the same institution. On the background of all these data, we have compared the recent averaged dosimetric results of 3D printed moulds with the averaged results published in our earlier study using conventional epithetic moulds [16] and identical prescription and fractionation concepts.

The first question was to quantify the dosimetric benefits of the advanced 3D printing design and manufacture process with respect to the conventionally manufactured surface moulds. The second question was to evaluate how the treated plans of the patients wearing the 3D printed moulds realise the intentions of the pre-plans that were used to design the moulds. This includes checking the consistency of the treated plans with the pre-plans with respect to PTV coverage and sparing of OARs (eyeballs and lenses). The third question was to countercheck the suitability of water-based clinical TG-43 [17] treatment planning using the non-water-equivalent mould applicators against retrospective tissue-, material- and density-based TG-186 [18] calculations applying Hounsfield unit (HU)-associated density information obtained from the CT data used for treatment planning [17, 18].

## Materials and methods

In our department, selected patients with facial skin cancer have been treated by HDR brachytherapy with individually adapted mould applicators since 2019. This modality was initially introduced with patient-individual moulds manufactured in cooperation with an epithetician and resulted in good dosimetric and clinical experience. The clinical workflow and results as well as dosimetric evaluations for a cohort of 6 patients have been published previously [8, 16]. Manual mould adaption was replaced by 3D printer-based mould production technology, which enabled a standardised efficient full in-house preparation and treatment process. In particular, the 3D printing technique allowed advanced pre-planning-based dosimetric mould optimisation and reliable final dose calculations with TG-43 formalism.

A collective of 42 facial skin cancer patients treated with HDR brachytherapy with customised 3D printed surface moulds in our department and their treatment plans were dosimetrically assessed in this study. In this patient cohort, the distance to the OARs is comparable to the situation in patients with conventionally manufactured moulds. 24 female and 18 male patients with a median age of 79 years were suffering from basal cell carcinoma, squamous cell carcinoma, lentigo maligna or melanoma. 38 patients were treated at a single lesion, and the other 4 patients were treated simultaneously at two, three or five lesions, respectively, with single moulds covering all PTVs.

**Fig. 1:**
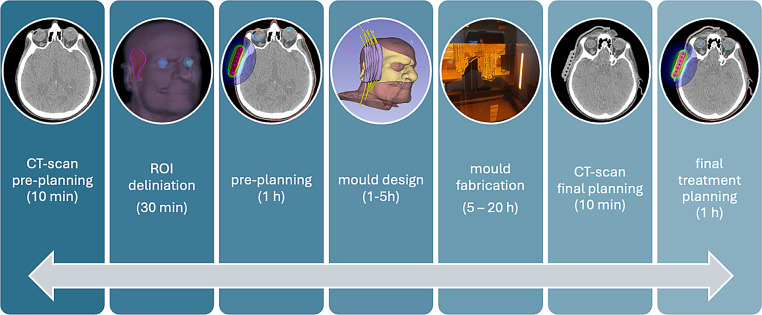
Graphical representation of the workflow for creating patient-individual 3D printed moulds. ROI region of interest

Depending on the diagnosis, the total PTV doses were typically prescribed as 30.0 to 45.0 Gy in daily fractions of 3.0 Gy. The dose was prescribed to the PTV of 3–6 mm tissue depth comprising the tumour and safety margins. The treated PTV volumes ranged from 0.8 to 19.2 cm^3^, with a mean volume of 6.7 cm^3^. The clinical goal was to cover 90 to 100% of the PTV volumes with the prescribed dose, to limit the PTV volume receiving more than 150% of the prescribed dose to a maximum of 30% and to spare the critical OARs. Generally, in our patient cohort, eye lenses and eyeballs needed to be spared as well as achievable. The moulds were fabricated, and treatment was planned and realised according to the workflow shown in Fig. 1. The key procedures are described in more detail below.

### Pre-planning workflow

Each patient had two computed tomography appointments associated with the pre-planning and the final planning process, respectively. In the first appointment, high-resolution scans covering the patient’s head with a 0.8-mm non-overlapping slice distance with 1024 × 1024 pixels were acquired with a Brilliance Big Bore Oncology CT scanner (Philips, Amsterdam, the Netherlands). Scan lengths and fields of view were defined large enough to enclose the patient’s head, including enough volume extension to cover the intended mould volume. The artefact-reduction technology OMAR (Philips) was applied to correct artefacts from non-removable dentures or implants. A second CT scan comprising the tumour region was acquired with a 1-mm radiopaque wire shaped as marker for the intended PTV boundaries. Both image sets were fused in the treatment planning software Oncentra® Brachy 4.5.3 (Elekta AB, Stockholm, Sweden) for delineation of the PTVs and OARs.

A preliminary plan was then drawn up that defined the geometry of the moulds and, in particular, the catheter guides in such a way that an optimal dose distribution (optimal dose coverage in the PTV with simultaneous maximum protection of OARs) was achieved. Depending on the PTV depth and localisation, the catheter-to-skin distance was set at between 3 and 6 mm of a corresponding 3D outer margin of the external patient contour under visual guidance. Favourable catheter planes were checked by rotating the coordinate system and planes to an orientation allowing masking of the PTV with a minimum number of equidistant parallel catheter paths separated by 3 to 8 mm without falling below the minimal allowed bending radius of the Flexible Implant Tube 6F (Elekta AB, Sweden) of 15 mm. Active dwell positions were defined to cover the PTV conformally, and the dose was optimised using the HIPO (hybrid inverse plan optimisation) module of Oncentra® Brachy focused on minimum and maximum PTV dose objectives of 100 and 150% of the prescribed dose. OAR objectives were applied to spare the eyeballs and lenses as well as achievable without underdosage of the PTV. The finalised pre-plans satisfying the clinical requirements were approved for further processing.

### 3D design and printing

Following the preliminary planning process, the preparatory processes for creating the moulds were carried out in 3D Slicer (open-source software, www.slicer.org). In the native CT dataset (without the marker wire in order to avoid artefacts), a structure for the external patient contour was generated using a thresholding method, analogous to the procedure described by Otsu et al. [19]. If necessary, artefacts impairing the facial surface were corrected to achieve the most precise reconstruction. A 5 mm thick shell was added to this structure. This shell was cropped to the area of the PTV and anatomical supports required for reproducible positioning of the mould. This structure served as the base carrier for the catheter guides.

To create the catheter guides, the support points of the catheter paths were extracted from the DICOM RT plan object and were transferred to 3D Slicer. These points were then connected to form paths using Kochanek–Bartel splines [20]. The paths were extended by a radius of 7.5 mm to form the catheter guides. The channels for commercially available interstitial 6F brachytherapy implant tubes are created by extending the paths by a radius of 1.5 mm and subtracting them from the catheter guides. The base carrier and the catheter guides were then joined together to form a complete patient-individual mould.

**Fig. 2:**
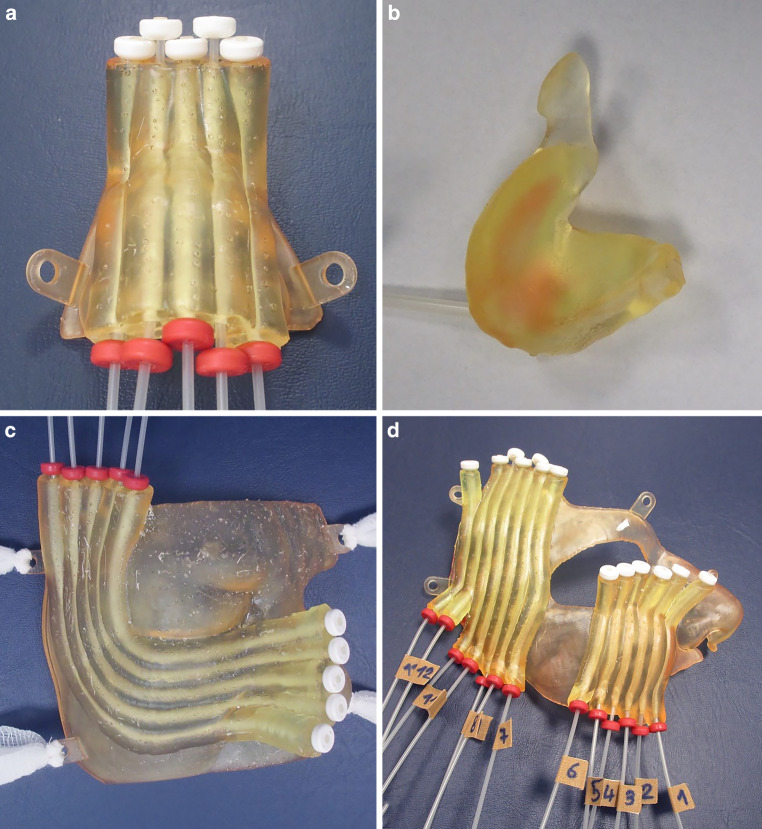
Four exemplarily 3D printed moulds for different tumour locations: **a** bridge of the nose; **b** external auditory canal with single source channel; **c,** **d** cheeks including lower limb and temple, respectively

The 3D structure of this mould was exported to Meshmixer (Autodesk Inc., San Francisco, CA, USA) for smoothing its edges. It was finally processed for stereolithography (SLA) 3D printing with PreForm (Formlabs Inc., Somerville, MA, USA). The moulds were fabricated with a Form 3 BL 3D printer for objects up to 33.5 × 20 × 30 cm^3^ with biocompatible resins for medical applications (Formlabs Inc.). The first 25 moulds were printed with rigid Dental SG and the later moulds were produced with the first generation of a flexible elastomer material IBT resin (both Formlabs Inc.) to further improve fit and comfort for patients. The brachytherapy implant tubes with radiopaque end buttons were pulled through the catheter paths and finally locked with opposite buttons. Figure 2 exemplarily shows four finalised moulds covering different tumour locations. For the tiny mould used inside an earlobe (Fig. 2b), a small amount of resin was used to fix the implant tube instead. All implant tubes were consecutively numbered for identification, and channel lengths were measured for final treatment planning.

### Final treatment planning workflow and treatment

At the second appointment for final treatment planning CT acquisition, the patient was positioned in the same way as for the pre-planning CT and was wearing the mould. Moulds were accurately positioned under visual control. Immediately after acquisition, the images were reviewed for correct fit of the mould. In rare cases (3 of 42 patients) of disadvantageous air gaps between patient and mould, the positioning and CT scan had to be repeated. In very rare cases (1 of 42 patients) with anatomically difficult locations, the mould had to be modified or redesigned for adequate fit, and these patients needed a third pre-treatment appointment.

**Fig. 3:**
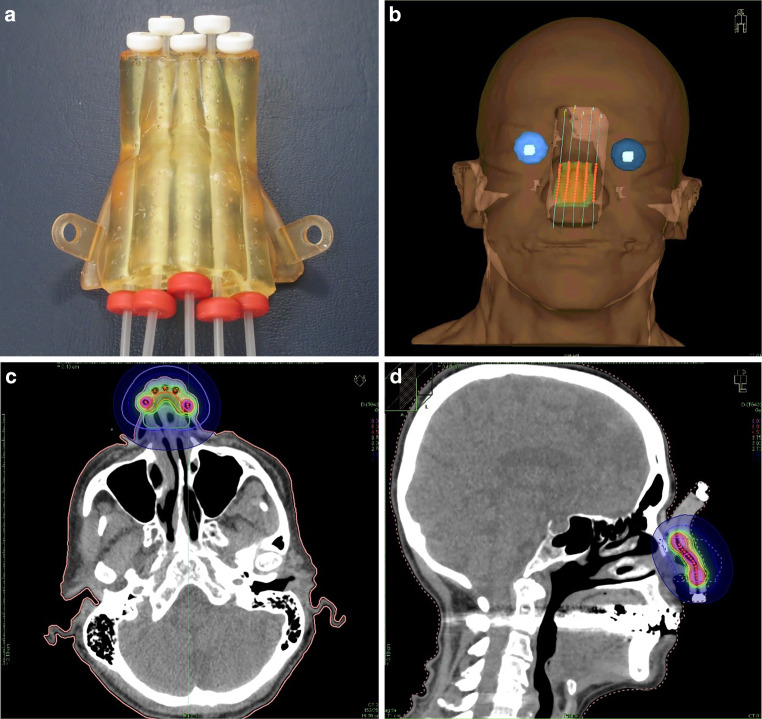
3D printed mould for definitive treatment of a squamous cell carcinoma on the bridge of the nose. 3D printed mould with 5 flexible implant tubes (**a**); 3D view of regions of interest, reconstructed catheters and activated source positions of the final treatment planning CT with mould (**b**); CT slices with relative isodose distributions in transverse and sagittal orientations of the planning target volume for the treated plan, TG-43 calculation (**c**, **d**)

**Fig. 4:**
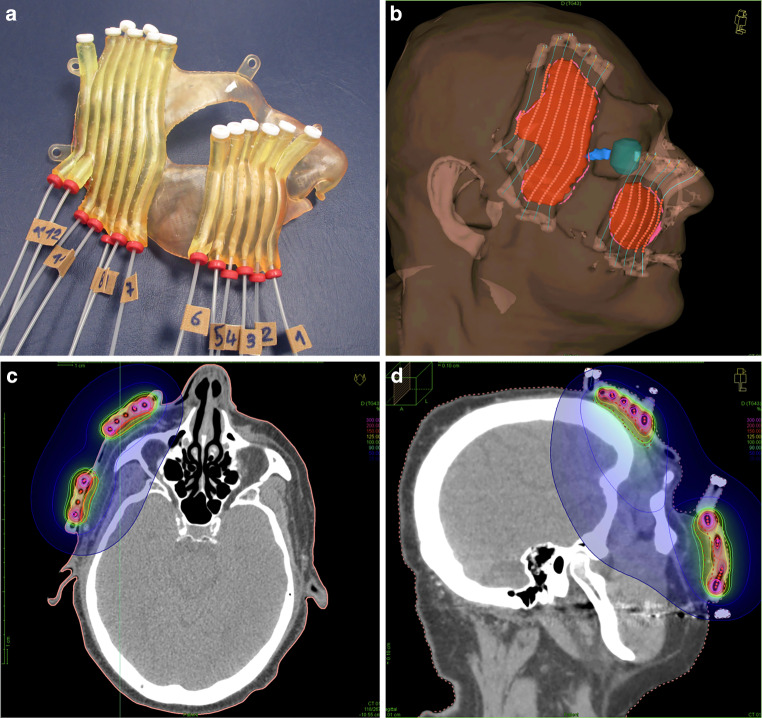
3D printed mould used for an additive treatment of the right cheek to lower limb (two planning target volumes [PTVs]) of a lentigo maligna after R1 resection. 3D printed mould with 13 flexible implant tubes (**a**); 3D view of regions of interest, reconstructed catheters and activated source positions of the final treatment planning CT with mould (**b**); CT slices with relative isodose distributions in transverse and sagittal orientations of the PTV for the treated plan, TG-43 calculation (**c**, **d**)

The final treatment planning CT data with the mould were registered to the pre-planning CT to transfer the structures to this dataset. The PTVs and OARs were checked, and irregularities caused by tilting of the corresponding slices were corrected. All catheter paths were reconstructed again to consider the exact positions from the new treatment planning CT. The initial active dwell positions and optimisation objectives were defined analogously to the pre-plan and were optionally adapted to reproduce the dosimetric quality of the pre-plan as well as achievable. After the inverse optimisation, the isodose coverage of the PTV was further optimised according to the clinical intentions by manual graphical isodose optimisation. Figures 3 and 4 show exemplary images of 3D printed moulds, reconstructed catheter paths with active dwell positions in 3D views and isodose visualisations of clinical treatment plans. Figure 3 depicts the definitive treatment of a squamous cell carcinoma (SCC) on the bridge of the nose. Fig. 4 shows the additive treatment of the right cheek to lower limb (two PTVs) of a lentigo maligna after R1 resection. Figure 4 shows that complex multiple PTV plans can be treated to the same high dosimetric standards as frequent single PTV plans of the nose, like that shown in Fig. 3.

### Dosimetric assessment

**Fig. 5:**
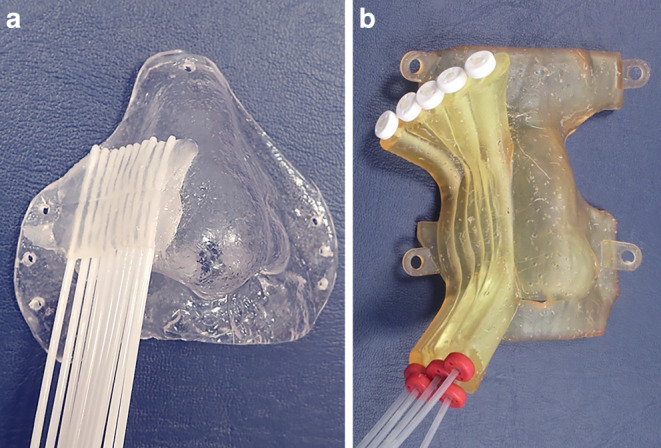
Conventionally fabricated epithetic mould (**a**) versus 3D printed mould (**b**) used to treat lesions of the right nasal wings for different patients

The key dose values achieved using 3D printed moulds were compared with those of our earlier treatments using conventionally fabricated moulds with the assistance of an epithetician [16]. These moulds were characterised by glued-on plastic needles on the surface, as shown in Fig. 5, covering the estimated tumour location according to the radiation therapist’s clinical observation. Neither were CT data available to manufacture these moulds, nor could pre-planning be used to optimise catheter positioning. Treatment planning was only possible based on the geometry specified by the epithetician.

The dosimetric outcomes of the clinically treated plans as well as the pre-plans used for 3D mould design were compared in terms of the PTV dose parameters D_90_, V_100_ and V_150_ and the OAR dose parameters (D_max_ and D_2cm³_ of the eyeballs and D_max_ of the eye lenses) for each individual patient. Average values and standard deviations were calculated for all examined parameters. The pre-plans as well as the treated plans were calculated with the classical TG-43 dose formalism [17], which is still the gold standard for clinical brachytherapy.

**Fig. 6:**
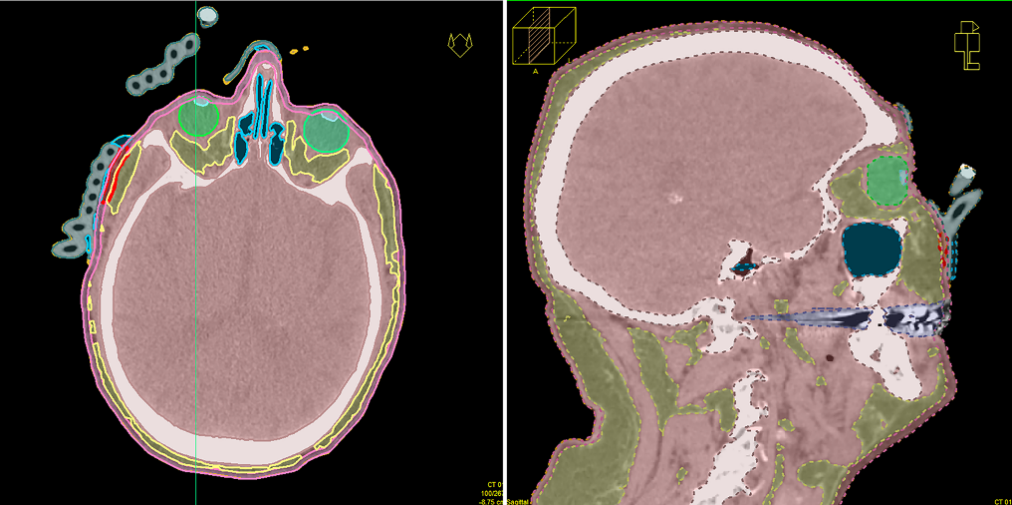
Example case showing all structures that were considered in the TG-186-based D_*m,m*_ dose calculations. The different tissue and material types were assigned according to our convention listed in Table 1 for all patient cases

For deeper dosimetric analysis, all treated plans (3D as well as conventional) were recalculated with the model-based ACE (advanced collapsed cone engine) of the latest Oncentra® Brachy 4.6.3 version, which enables tissue- and material-type-related D_*m,m*_ dose calculations according to the TG-186 formalism [18]. For this purpose, all dosimetrically relevant structures were retrospectively delineated. Table 1 shows the tissue and material types assigned to the structures in highest (bone) to lowest (external contour) priority ranking order. In the case of overlapping structures, the higher-prioritised structure is decisive. Dosimetrically relevant metal artifacts originating from non-removable dentures were delineated as separate substructures of the underlying tissue types with higher priority and were assigned with their uniform tissue-related mass densities. The PTV itself does not represent a specific tissue in the strict sense and is solely used for dose statistical purpose, so no tissue type should be assigned. There are no specific material types in Oncentra Brachy for the 3D mould materials used. Based on the chemical composition of the photopolymer resins Dental SG and IBT, PMMA was identified as the best available material type. As the conventional moulds consisted of similar thermoplastic material, PMMA was also assigned to these epithetic moulds. An example case with all contours is shown in Fig. 6.

**Table 1:** Tissue and material types assigned for the advanced collapsed cone engine calculations in order of highest to lowest priority. All structures were manually delineated unless otherwise specified

Priority of assignment	ROI	DICOM type	Tissue or material type	Delineation convention
Highest	Bone	Avoidance	Cortical bone	Automatic contouring, manually corrected
↓	Left eye lens	Avoidance	Eye lens	Anatomical
Lowest	Right eye lens	Avoidance	Eye lens	Anatomical
	Left eyeball	Avoidance	Water	Anatomical
	Right eyeball	Avoidance	Water	Anatomical
	Air	Patient cavity	Air	Air cavities inside external contour
	Fat	Avoidance	Mean adipose	Anatomically visible structures (orbital cavity, subcutaneous fat)
	Skin	Avoidance	Mean skin	Epidermis and dermis, automatic contouring, 3‑mm inner margin from the patient contour
	Patient contour	Avoidance	Female or male soft tissue	Patient head only
	Mould	Brachy source applicator	PMMA	Mould volume including end buttons
	External contour	External	–	Patient head and mould, automatic contouring, manually corrected
Higher than underlying structure	Artefacts	Previously tissue type	Uniform mass densities	CT slices with metal artefacts
n. a.	PTV	Planning target volume	–	Tumour volume with margins

In general, all tissue and material densities were calculated with HU-based mass densities derived from the CT scans, which consider individual patient-specific scattering and attenuation of the patient and mould as well as the surrounding air. With this calculation principle, D_*m,m*_ of each calculated voxel of the dose matrix depends on the electron density of the associated tissue or material type as well as the local HU-derived mass and electron density [21, 22]. All ACE calculations were performed at the “high accuracy” level and were documented as post-plans in the “Results” section.

## Results

All patients could be treated completely according to the initial prescriptions. Some of them were treated with hypofractionation every other day for their last 5 to 10 fractions to stabilise acute radiation dermatitis. The therapeutic success and observed toxicity of the first 15 patients were described by Chatzikonstantinou et al. [9]. For every treated fraction, the mould was positioned exactly as for the treatment planning CT acquisition, and the achieved reproducibility was always good under visual control.

Dosimetric outcomes with respect to PTV coverage and OAR exposure were statistically monitored for all 48 patients included in the study (42 patients treated with 3D printed moulds, 6 patients with conventionally fabricated moulds). The pre-plans as well as clinical treatment plans were compared by considering the relative PTV dose parameters D_90_, D_98_, D_100_, V_100_ and V_150_ as well as the absolute OAR dose parameters of all fractions (D_max_ and D_2cm³_ of the eyeballs and D_max_ of the eye lenses) for each patient.

**Fig. 7:**
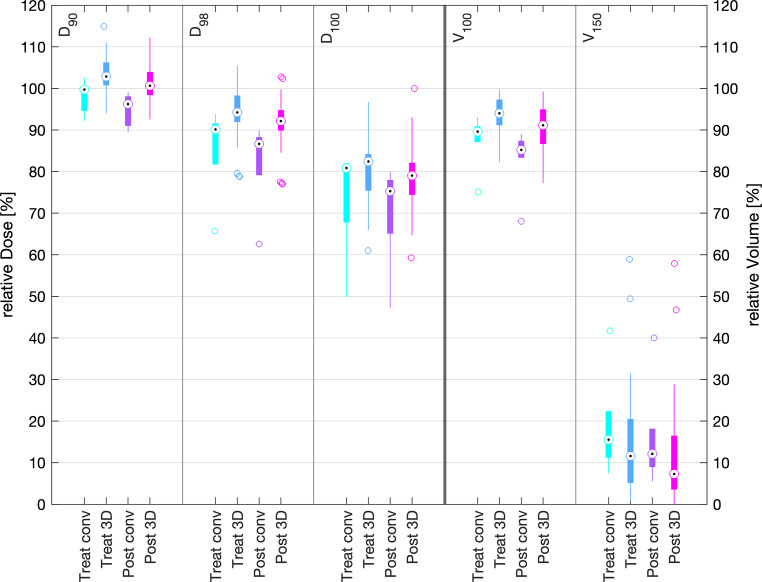
Comparison of planning target volume dose parameters of the clinically applied TG-43 plan (*Treat*) and the retrospective TG-186 calculated plans (*Post*) for conventional (*conv*) and 3D-printed (*3D*) moulds. The black dots represent the median values, the coloured boxes represent the intervals of the mean 50% percentile data points (interquartile range), the whiskers represent 1.5 times the interquartile range (extreme data points outsides this range are shown as separated circles)

**Fig. 8:**
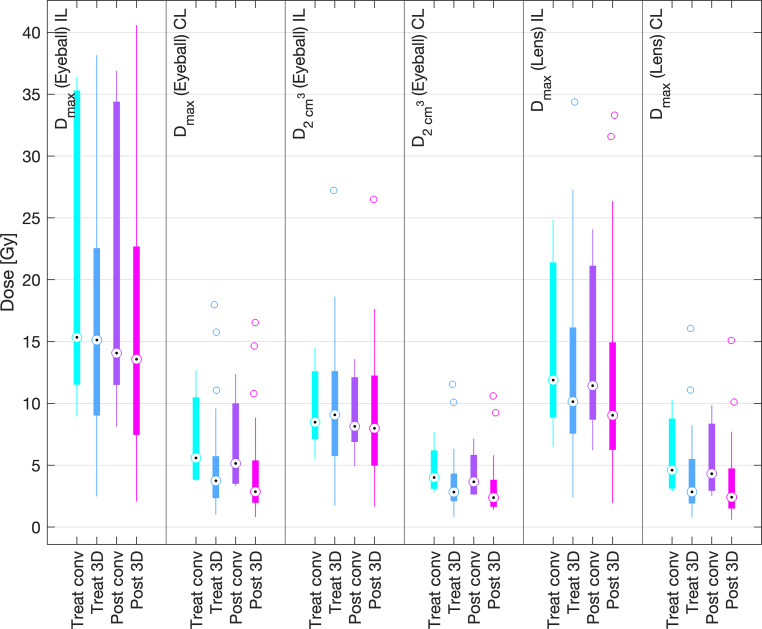
Comparison of the dose parameters of the treated TG-43 plans (*Treat*) and the retrospectively recalculated TG-186 doses (*Post*) of the risk organs (eyeballs and lenses) during irradiation with conventional (*conv*) and 3D printed (*3D*) moulds

The boxplots shown in Figs. 7 and 8 compare the dosimetric results of the 42-patient cohort treated with 3D printed moulds with the data of the 6‑patient cohort previously treated with conventional epithetic moulds [16]. This first direct dosimetric comparison of two mould-manufacturing techniques comprises the TG-43- (“Treat”) and TG-186-calculated (“Post”) doses of both cohorts. The 42 patients treated with the 3D mould were characterised by a mean PTV volume of 6.7 cm^3^ (range 0.8 cm^3^–19.2 cm^3^) treated with a mean of 6.5 (range 1–17) flexible brachytherapy catheters, whilst the epithetic mould patients were characterised by 4.3 cm^3^ mean PTV volume (range 1.8 cm^3^–6.8 cm^3^) and treated with a mean of 11.8 (range 6–18) brachytherapy catheters.

Dosimetric TG-43 mean values of the treated plans were D_90_ = 104.3 ± 4.8%, V_100_ = 94.4 ± 3.8% and V_150_ = 14.0 ± 12.5% for 3D mould plans versus D_90_ = 98.2 ± 3.7%, V_100_ = 87.6 ± 5.9% and V_150_ = 19.0 ± 11.2% for the epithetic mould plans. Dosimetric TG-186 mean values of the treated plans were D_90_ = 101.1 ± 4.2%, V_100_ = 90.5 ± 5.4% and V_150_ = 11.8 ± 11.9% for 3D mould plans versus D_90_ = 95.0 ± 3.6%, V_100_ = 83.0 ± 7.0% and V_150_ = 16.2 ± 11.4% for the epithetic mould plans.

TG-43 mean values of D_max_ of the ipsilateral and contralateral eyeballs with 3D printed moulds were 16.2 ± 9.7 Gy and 5.0 ± 1.8 Gy, and mean D_max_ of the ipsilateral and contralateral lenses was 12.4 ± 8.4 Gy and 4.2 ± 1.7 Gy. For conventional moulds, mean values of D_max_ of the ipsilateral and contralateral eyeballs were 20.5 ± 11.1 Gy and 7.0 ± 3.5 Gy, and mean D_max_ of the ipsilateral and contralateral lenses was 14.2 ± 6.8 Gy and 5.7 ± 2.9 Gy, respectively. The recalculation according to TG-186 resulted in mean values of D_max_ of the ipsilateral and contralateral eyeballs with 3D printed moulds of 15.6 ± 10.3 Gy and 4.2 ± 3.4 Gy, respectively. Mean D_max_ of the ipsilateral and contralateral lenses was 11.4 ± 7.8 Gy and 3.5 ± 2.8 Gy, respectively. D_max_ of the ipsilateral and contralateral eyeballs with conventional moulds was 19.8 ± 11.4 Gy and 6.6 ± 3.5 Gy, and mean D_max_ of the ipsilateral and contralateral lenses was 13.8 ± 6.6 Gy and 5.4 ± 2.9 Gy, respectively.

**Fig. 9:**
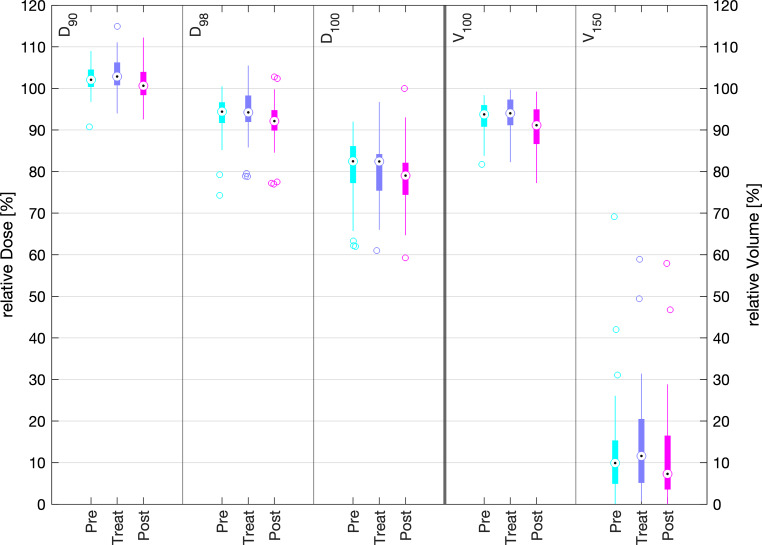
Boxplots of planning target volume dose parameters for 3D printed moulds of the TG-43 preplan (*Pre*), the clinically applied TG-43 plans (*Treat*) and the retrospective TG-186-calculated plans (*Post*) of all 42 patients included in the study

**Fig. 10:**
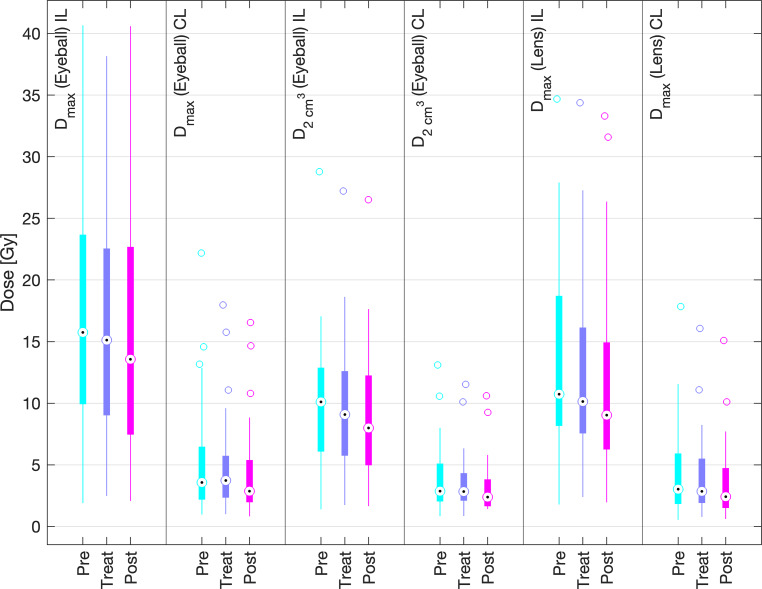
Comparison of the organs at risk (eyeballs and lenses) dose parameters with 3D printed moulds in the TG-43 preplan (*Pre*), the clinically applied TG-43 plan (*Treat*) and the retrospective TG-186-calculated plans (*Post*) of all 42 patients included in the study

Boxplot visualisations of all patients treated with 3D moulds concerning the PTV data are shown in Fig. 9 and concerning the OAR data in Fig. 10. The initial parameters for the pre-plans (“Pre”) used to design the moulds are shown in the left plots of each column of Fig. 9. Regarding the averaged D_90_ = 102.4 ± 1.3, V_100_ = 92.9 ± 2.1% and V_150_ = 11.5 ± 8.5%, these values coincided well with the clinical goals of D_90_ > 100%, V_100_ > 90% and V_150_ < 30% and the standard deviations were even lower than for the treated plans (middle plots of Fig. 9, “Treat”). While the preliminary plans were HIPO optimised based exclusively on cost functions, the treated plans were further adapted by clinically oriented graphical optimisation before approval by the radiation therapist. The averaged D_90_ of 104.3 ± 4.8%, V_100_ of 94.4 ± 3.8% and V_150_ of 14.0 ± 12.5% and the standard deviations of the treated plans show that the dosimetric goals of the preliminary plans are mainly reproduced in the treated plans. Average D_98_ > 90% and D_100_ > 80% statistics for the optimised pre-plans and the treated plans underscore the advantageous PTV coverage of the pre-plan-based 3D printed moulds and the good reproducibility of the pre-plan results in the final treated plans.

The middle and right plots of each column in Fig. 9 compare the TG-43-calculated doses of the treated plans (Treat) and their re-calculated (not re-optimised) TG-186 equivalents (Post). The latter plans considered patient-individual CT-derived density matrices (HU-based density association) in the ACE dose engine. Generally, the classic TG-43-calculated doses assuming a water environment were slightly higher than the TG-186-calculated doses. Concerning all PTV parameters, the differences in the mean values for TG-43 and TG-186 were less than 3.9 percentage points, i.e. D_90_ = 101.1 ± 4.2%, V_100_ = 90.5 ± 5.4% and V_150_ = 11.8 ± 11.9% for the TG-186 post-plans.

Figure 10 depicts the same evaluation for the organs at risk. D_max_ values of the ipsilateral and contralateral eyeballs were 16.2 ± 9.7 Gy and 5.0 ± 1.8 Gy, respectively. D_max_ values of the ipsilateral and contralateral lenses were 12.4 ± 8.4 Gy and 4.2 ± 1.7 Gy, respectively, for the clinical TG-43 calculations (Treat). Concerning the pre-plans (Pre), D_max_ of the ipsilateral and contralateral eyeballs was 16.3 ± 10.0 Gy and 4.8 ± 1.8 Gy, respectively, and D_max_ of the ipsilateral and contralateral lenses was 12.7 ± 8.4 Gy and 4.0 ± 1.6 Gy, respectively. The TG-186 equivalents show the same slightly lower doses, with D_max_ values of 15.6 ± 10.3 Gy and 4.2 ± 3.4 Gy for the ipsilateral and contralateral eyeballs and 11.4 ± 7.8 Gy and 3.5 ± 2.8 Gy for the ipsilateral and contralateral lenses, respectively.

## Discussion

Individual 3D printed moulds have surpassed the status of feasibility studies and are now established in our clinical routine. The stereolithography (SLA) 3D printing method is a technique that generates very homogenous and true-to-scale objects that are typically free of systematic air inclusions like it is observed for fused material deposition (FDM) 3D printing. Thus, the processed Dental SG and IBT photopolymer moulds appear as homogeneous objects in our CT images, i.e. CT density is clearly defined and reproducible. The whole 3D manufacturing process can be established in house, without any external service provider involved in mould production, as was required for the epithetic moulds used before. A total of 4 to 12 working hours per mould must be estimated for the current workflow. This time effort may be reduced in future by automated or at least assisted catheter path routing and geometric mould generation with dedicated software or automatic contouring and scripting integrated in advanced treatment planning systems.

The dosimetric evaluation underscores the fact that the PTV-related clinical goals intended from the pre-plans were reproduced with the treated plans within the standard deviations. The dosimetric results of the 3D printed moulds show better PTV coverage and dose conformity according to the prescription than the results of the conventional epithetic moulds. For example, the average D_90_ for the PTV improved by 6 percentage points from 98.25 to 104.30%. This proves the benefit of patient-individual pre-planning within the HDR brachytherapy workflow using 3D printed patient-individual moulds, the most important step of the workflow to guaranty the intended dose–volume coverage. Even though our conventional mould-treated patients typically had smaller PTV volumes and the epithetic moulds were equipped with more brachytherapy catheters or plastic needles than the 3D printed moulds, all clinical goals were better satisfied (i.e. closer to the intentions) and more reproducible (i.e. lower standard deviations) with the 3D printed moulds.

High-dose restrictions for the organs at risk were considered in the pre-plan optimisations as well as in the final treatment plan optimisations. For most of our patients the doses to the lenses or at least to one lens could be restricted to acceptable values with a low risk for cataracts. The maximum dose to the contralateral eyeball and the contralateral lens could be reduced by up to 30% compared to the treatments with conventional moulds. In addition, the range of dose exposure to the organs at risk was significantly reduced. Thus, the average values shown in Fig. 10 (Treat) are characterised by a wide range for the patient collective, which results in larger standard deviations than for the PTV parameters due to a variety of tumour localisations.

In summary, the availability of preliminary planning when creating 3D moulds provides the following advantages compared to conventionally produced moulds, where no preliminary planning is available:

- individual situation-adapted catheter orientations and routing based on the PTV and OAR localisations,
- optimisation of PTV dose coverage and OAR distances according to these catheter orientations,
- best achievable PTV coverage with the lowest possible number of brachytherapy catheters,
- interactive dwell position activation and inverse dose optimisation prior to mould production to test and improve the therapeutic potential in advance.

Recalculation of the dose distribution with the algorithm according to TG-186 shows a 3.9 percentage points difference compared to TG-43. As these values are still in line with the clinical goals on average, this is therefore of limited clinical relevance.

The relative electron densities for the IBT resin (1.08) and Dental SG resin (1.13) are within the same range or just moderately higher than for mean adipose tissue (0.95), water (1.00), mean female and male soft tissue (1.02, 1.03), and skin (1.09) [18]. It can be assumed that the slightly higher radiation interaction and backscattering in the mould material partially compensates for the reduced backscattering effect of the superficial treatment geometry. The comparison of ACE and TG-43 results reveal no significant lack of backscattering that principally concerns the superficial treatment situation [23]. This effect was sufficiently reduced with the more voluminous 3D printed moulds with respect to the previously manufactured epithetic moulds with less backscattering material. Consequently, for the PTV, the TG-186 versus TG-43 differences were lower for the 3D printed moulds than for the epithetic moulds. Furthermore, the epithetic mould materials (support layer and resin glue) were affected by inhomogeneous air inclusions resulting in an inhomogeneous HU voxel matrix [16].

In conclusion, the model-based dose calculation ACE algorithm is helpful for evaluation of the dosimetric correctness of the 3D mould-related treatment planning but is not a requirement for adequate clinical application. Several authors have already validated the suitability of TG-43 dose calculations in relation to FDM 3D printed moulds using different plastic materials, like PLA, ABS and others [24–28]. The fast and reliable TG-43 formalism is favourable for creating the clinical treatment plans of 3D printed moulds, and model-based dose calculation algorithms are helpful for evaluation of dosimetric correctness but are, in most situations, not a precondition for routine clinical application.

## Conclusion

Customised 3D printed moulds offer a safe and efficient technique for the treatment of facial skin cancer with HDR brachytherapy in critical locations and complex clinical situations. In direct comparison, dosimetric outcomes and interfractional position reproducibility with 3D printed moulds are superior to conventionally manufactured facial moulds with respect to the clinical goals. The two-step planning process results in reliably improved PTV dose coverage, with an increase in D_90_ for the PTV from 98.3% for the 6‑patient cohort treated with conventionally fabricated moulds to 104.3% for the 42-patient cohort treated with 3D printed moulds, and more efficient sparing of eye lenses and eyeballs, with a reduction of up to 30% of the D_max_ values. No clinically decisive deviations of TG-43 and HU-based TG-186 calculations were observed with the 3D printed moulds. The fast and reliable planning with the commonly used TG-43 formalism is sufficient for treatment with 3D printed patient-individual moulds made of IBT and Dental SG resin in HDR brachytherapy for skin cancer.
